# Repeated misdiagnosis of small intestine bronchogenic cyst: a case report

**DOI:** 10.3389/fonc.2024.1259335

**Published:** 2024-01-23

**Authors:** Xiaodong Chen, Danfei Hu, Wenbo Gao, Qihang Wu, Xiangcheng Qin, Zhichao Wang, Yangkai Xu, Dong Chen, Nan Li, Guobin Weng

**Affiliations:** ^1^ Department of Urology, Ningbo Urology and Nephrology Hospital, Ningbo, Zhejiang, China; ^2^ Department of Radiation Therapy, Ningbo Medical Treatment Center Lihuili Hospital, Ningbo, Zhejiang, China

**Keywords:** small intestine bronchogenic cyst, misdiagnosis, case report, surgery, ectopic bronchogenic cyst

## Abstract

Bronchogenic cysts are uncommon congenital malformations of the respiratory system. These cysts can be categorized as intrapulmonary, mediastinal, or ectopic. Ectopic bronchogenic cysts, which lack distinctive clinical and imaging features, are particularly challenging to diagnose. This study presents a 48-year-old woman having a small intestinal bronchogenic cyst. She was repeatedly misdiagnosed as having an ovarian chocolate cyst or a cystic mass of bladder origin three years ago. However, no cyst was found during the operation. Half a year prior to presenting at our hospital, the patient developed frequent urination, prompting her to seek further treatment. We eventually discovered a cyst in the small intestine. The histological evaluation of the specimen showed a bronchogenic cyst. Small intestine bronchogenic cysts are extremely rare and easily misdiagnosed. It should be considered as one of the differential diagnoses of pelvic cysts. Particularly, when intraoperative exploration of the pelvic cavity fails to detect any cysts, consideration should be given to the possibility of small intestine bronchogenic cysts.

## Introduction

Bronchogenic cysts are uncommon congenital malformations of the respiratory system. The exact mechanism of their development remains unclear, but current research suggests that abnormal budding of the ventral diverticulum of the foregut during embryonic development between the 26th and 40th days of gestation is the primary cause (1). These cysts can be categorized as intrapulmonary, mediastinal, or ectopic. Ectopic bronchogenic cysts, which lack distinctive clinical and imaging features, are particularly challenging to diagnose. They are typically discovered incidentally during routine physical examinations or when complications arise due to compression of adjacent organs or tissues (2).

However, there have been limited reports of bronchogenic cysts occurring in the small intestine. In this case report, we present a patient with a small intestinal bronchogenic cyst that was repeatedly misdiagnosed.

## Case presentation

**Figure 1:**
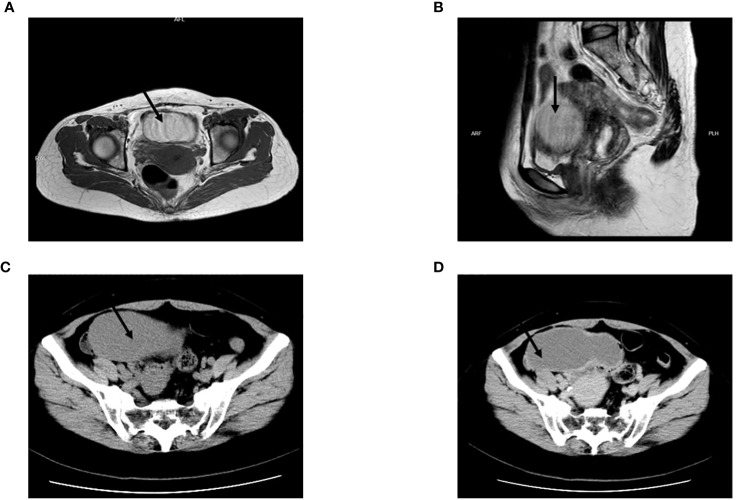
**(A)** Contrast-enhanced MRI of the pelvis revealed a circular cyst measuring 8 × 5 × 6 cm located in front of the uterus. The cyst exhibited T1 hypersignal. **(B)** The cyst exhibited T2 slightly hypersignal. **(C)** CTU revealed a cyst measuring 9.9 × 5.7 cm in the right pelvic cavity, exhibiting a CT value of approximately 39 HU. **(D)** The cyst exhibited a clear and smooth edge, with enhancement observed during the excretion phase of the CTU, while the solid portion showed minimal enhancement.

**Figure 2:**
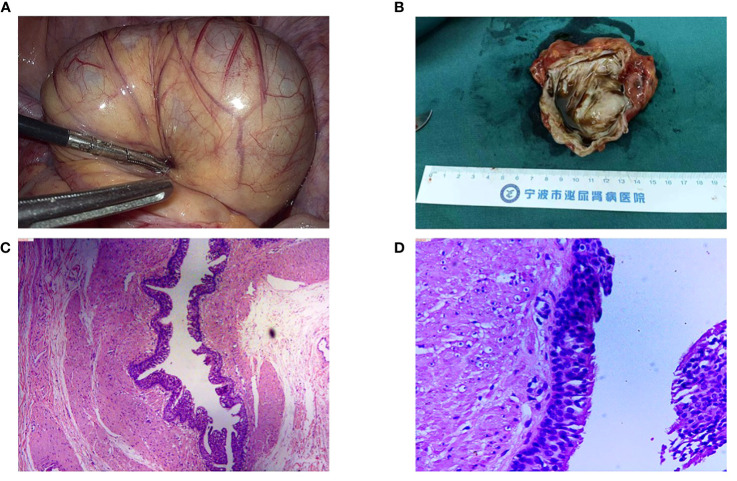
**(A)** We discovered a cyst in the small intestine during the surgery. **(B)** The cyst lesion originated from the small intestine. Sectioning revealed brown and sticky fluid. **(C)** Histological evaluation of the specimen showing the presence of ciliated columnar epithelium on its surface, original magnification, 50×. **(D)** Original magnification, 200×.

A 48-year-old female patient presented with a three-year history of pelvic cyst detection and experiencing frequent urination for the past six months. The initial detection of the pelvic cyst occurred during a medical examination three years ago, after which the patient sought treatment at a local Women’s and Children’s Hospital. Subsequently, the patient underwent a contrast-enhanced MRI of the pelvis. The MRI results revealed a circular cyst measuring 8 × 5 × 6 cm located in front of the uterus. The cyst exhibited T1 hypersignal (**Figure 1A**) and slightly hypersignal on T2 (**Figure 1B**), with no significant enhancement observed following the enhanced scan. Initially, the cyst was suspected to be an ovarian chocolate cyst, leading to laparoscopic exploration. However, no ovarian cyst was found during the operation. Subsequently, an interhospital consultation was requested, and a urologist from another hospital suggested the cyst might have originated from the bladder. Despite surgical exploration being performed again, the cyst remained elusive, and it was presumed to have disappeared. Half a year prior to presenting at our hospital, the patient developed frequent urination, prompting her to seek further treatment. The patient had no notable personal or family medical history, and the physical examination did not reveal any abnormalities. The laboratory results indicated only an elevation in the serum tumor marker CA199 (107.3 U/mL; normal reference range: <37 U/mL). Other parameters such as liver and kidney function, blood routine, and urine routine fell within normal ranges. CTU revealed a cyst measuring 9.9 × 5.7 cm in the right pelvic cavity, exhibiting a CT value of approximately 39 HU (**Figure 1C**). The cyst exhibited a clear and smooth edge, with enhancement observed during the excretion phase of the CTU, while the solid portion showed minimal enhancement (**Figure 1D**). The presence of the cyst pressing on the bladder was considered as the cause of frequent urination. Subsequently, laparoscopic resection of the pelvic cyst was performed, but no cyst was found in the pelvic cavity. Cavity and intraoperative ultrasound examination did not indicate the presence of a pelvic cyst. To our surprise, a cyst was discovered in the small intestine during the surgery, prompting its removal (**Figure 2A**). The cyst lesion originated from the small intestine. Sectioning revealed brown and sticky fluid (**Figure 2B**). Histological evaluation of the cyst revealed the presence of ciliated columnar epithelium on its surface, indicating a bronchogenic cyst (**Figures 2C, D**).

## Discussion

Bronchogenic cysts are exceedingly rare, with an incidence of approximately 1 in 68,000 to 1 in 42,000. They are predominantly located in the mediastinum, although they can also occur in other sites such as the lung parenchyma, pleura, and diaphragm (3–5). Ectopic bronchogenic cysts are even rarer and have been reported in various locations, including the skin, subcutaneous tissue, neck, mesentery, and intramedullary part of the spine (6).

The diagnosis of bronchogenic cysts typically involves a combination of ultrasound, CT, and MRI. On ultrasound, bronchogenic cysts typically present as cystic masses. CT scans reveal well-defined, homogeneous masses with low density. The attenuation coefficients on CT can vary based on the contents of the cyst, with higher protein or calcium oxalate levels resulting in increased attenuation (7). MRI is considered superior to CT for diagnosing bronchogenic cysts (8). In T1-weighted sequences, the signal intensity of bronchogenic cysts varies depending on their contents, while in T2-weighted sequences, they typically exhibit high signal intensity (6, 9). Intraabdominal bronchogenic cysts are extremely rare and are usually located in the left to midline and near the spleen, pancreas tail, and left adrenal gland (10, 11). The CT scan showed the pancreas bronchogenic cyst as a well-defined, non-enhancing, high-attenuated cyst (10). The main clinical manifestations of a gastric bronchogenic cyst are epigastric pain, nausea, and vomiting. The cyst appears on CT as a solitary, well-defined, low-density, homogeneous circular or oval cystic lesion attached to the stomach (7, 12). A report on the ileal bronchogenic cyst showed it to be a cystic mass with a complete capsule in abdominal enhanced CT (13). There are few reports on the imaging features of small intestine bronchogenic cyst. We found that small intestine bronchogenic cyst has no characteristic imaging features, which is one of the reasons for its repeated misdiagnosis. The final diagnosis still depends on pathology.

The surgical treatment of bronchogenic cysts remains a topic of debate, but most experts recommend surgical resection even in the absence of symptoms due to potential complications such as infection, rupture, intracapsular bleeding, and the risk of malignancy (14, 15).

In our case report, the patient was repeatedly misdiagnosed as having an ovarian chocolate cyst or a cystic mass of bladder origin. However, no lesions were found during the surgeries, and the cyst was initially presumed to have disappeared. Several factors contributed to this misdiagnosis: 1) The patient’s surgical position, typically with the head lower than the feet, caused the cyst to shift into the abdominal cavity along with the small intestine, making it difficult to locate during the operation; 2) Bronchogenic cysts lack specific clinical and imaging features, posing challenges for preoperative diagnosis; 3) Small intestine bronchogenic cysts are extremely rare and have been scarcely reported in the literature, leading to a lack of awareness among some medical professionals. Through this case report, we aim to enhance doctors’ understanding of this condition and emphasize the importance of distinguishing pelvic cysts from small intestine bronchogenic cysts. Particularly, when intraoperative exploration of the pelvic cavity fails to detect any cysts, consideration should be given to the possibility of small intestine bronchogenic cysts.

## Data availability statement

The original contributions presented in the study are included in the article/supplementary material. Further inquiries can be directed to the corresponding author.

## Ethics statement

The studies involving humans were approved by The Ethics Committee of Ningbo Urology and Nephrology Hospital. The studies were conducted in accordance with the local legislation and institutional requirements. Written informed consent for participation was not required from the participants or the participants’ legal guardians/next of kin in accordance with the national legislation and institutional requirements. Written informed consent was obtained from the individual(s) for the publication of any potentially identifiable images or data included in this article.

## Author contributions

XC: Writing – original draft. DH: Writing – original draft. WG: Investigation, Writing – original draft. QW: Investigation, Writing – original draft. XQ: Writing – original draft. ZW: Data curation, Writing – original draft. YX: Writing – original draft. DC: Writing – original draft. NL: Writing – original draft. GW: Supervision, Writing – review & editing.
